# Beyond Urticaria: Acute Airborne Contact Dermatitis in a Hospital Worker Presenting to the Emergency Department

**DOI:** 10.7759/cureus.99279

**Published:** 2025-12-15

**Authors:** Sreejith Jayachandran, Jobin Jose, Rebecca Paulose, Vinaya M Mathew, Mohammed Rizwan

**Affiliations:** 1 Emergency Medicine, All India Institute of Medical Sciences, Rishikesh, Rishikesh, IND; 2 Emergency Medicine, Amala institute of Medical Sciences, Thrissur, IND; 3 Emergency Medicine, Amala Institute of Medical Sciences, Thrissur, IND

**Keywords:** acute eczematous dermatitis, airborne allergic contact dermatitis, emergency department presentation, occupational exposure, quaternary ammonium compounds

## Abstract

Airborne allergic contact dermatitis (ACD) is a delayed, T-cell-driven inflammatory reaction that usually develops gradually in occupational settings, so encountering an acute presentation in the emergency department is uncommon. Severe, sudden exacerbations mimicking acute allergic emergencies are uncommon and can complicate emergency department (ED) evaluation. We describe a 45-year-old hospital cleaning staff member with a five-year history of intermittent dermatitis who presented with abrupt, diffuse pruritus, marked facial swelling, and vesiculopapular eruptions shortly after working with aerosolized quaternary ammonium disinfectants. Despite the dramatic cutaneous involvement, her vital signs remained within normal limits, distinguishing the presentation from anaphylaxis or widespread urticaria. Dermatologic examination demonstrated acute eczematous inflammation overlying chronic lichenified plaques, predominantly involving exposed areas. The patient was treated with a loading dose of intravenous methylprednisolone followed by scheduled systemic steroids, dual H1/H2 blockade, and potent topical corticosteroids, achieving rapid symptomatic improvement. This case reinforces the need to consider acute occupational ACD in the ED when intense pruritus and angioedema-like swelling occur without systemic compromise, and highlights the importance of pattern recognition and long-term avoidance counselling.

## Introduction

Contact dermatitis (CD) represents a common inflammatory skin disorder encountered in outpatient, occupational, and acute care settings. Among its subtypes, allergic contact dermatitis (ACD) is a delayed, T-lymphocyte-driven reaction that contributes substantially to work-related dermatologic disease worldwide [1]. A distinct and diagnostically challenging form is airborne contact dermatitis (ABCD), in which dispersed allergenic particles settle on exposed cutaneous surfaces. Because of its distribution and potentially widespread involvement, ABCD is prone to misinterpretation as other dermatoses [2].

Airborne exposure is frequently encountered in occupations involving aerosols, disinfectant sprays, dust, and volatile compounds. Within healthcare environments, cleaning personnel are repeatedly exposed to concentrated disinfectant agents, particularly quaternary ammonium compounds (QACs). Although chronic ABCD typically produces eczematous plaques, scaling, and lichenification in areas not covered by clothing, symptoms may suddenly intensify following heavy allergen exposure. A sudden increase in airborne allergen load can trigger a dramatic flare. Marked facial swelling and severe pruritus may resemble an acute allergic emergency, leading patients to seek urgent care.

This case describes a severe acute-on-chronic ABCD flare in a hospital cleaner and outlines the clinical indicators, diagnostic reasoning, and therapeutic approach that aided differentiation from anaphylaxis and generalized urticaria. The patient achieved complete resolution with systemic steroids and dual H1/H2 antihistamine therapy, with symptoms fully resolving by 15-day follow-up. This case is distinctive because airborne contact dermatitis rarely presents abruptly in the emergency department and may be mistaken for anaphylaxis. The dramatic flare followed heavy occupational exposure to aerosolized quaternary ammonium disinfectants, an increasingly recognized allergen in healthcare settings.

## Case presentation

A 45-year-old female hospital cleaner with a five-year history of intermittent dermatitis presented to the emergency department (ED) with severe, generalized pruritus and a rapidly spreading rash that began approximately four hours earlier. Symptoms developed during a work shift involving extensive use of aerosolized QAC disinfectants. She initially experienced burning, followed by escalating itch (10/10 on visual analogue scale) and noticeable facial swelling. She denied dyspnoea, dysphonia, chest tightness, dizziness, or presyncope.

On arrival, she was alert but markedly uncomfortable. Vital signs demonstrated hemodynamic stability: heart rate 88 beats/minute, blood pressure 130/85 mmHg, and oxygen saturation 98% on room air. The absence of respiratory or cardiovascular involvement lowered suspicion for anaphylaxis.

**Figure 1:**
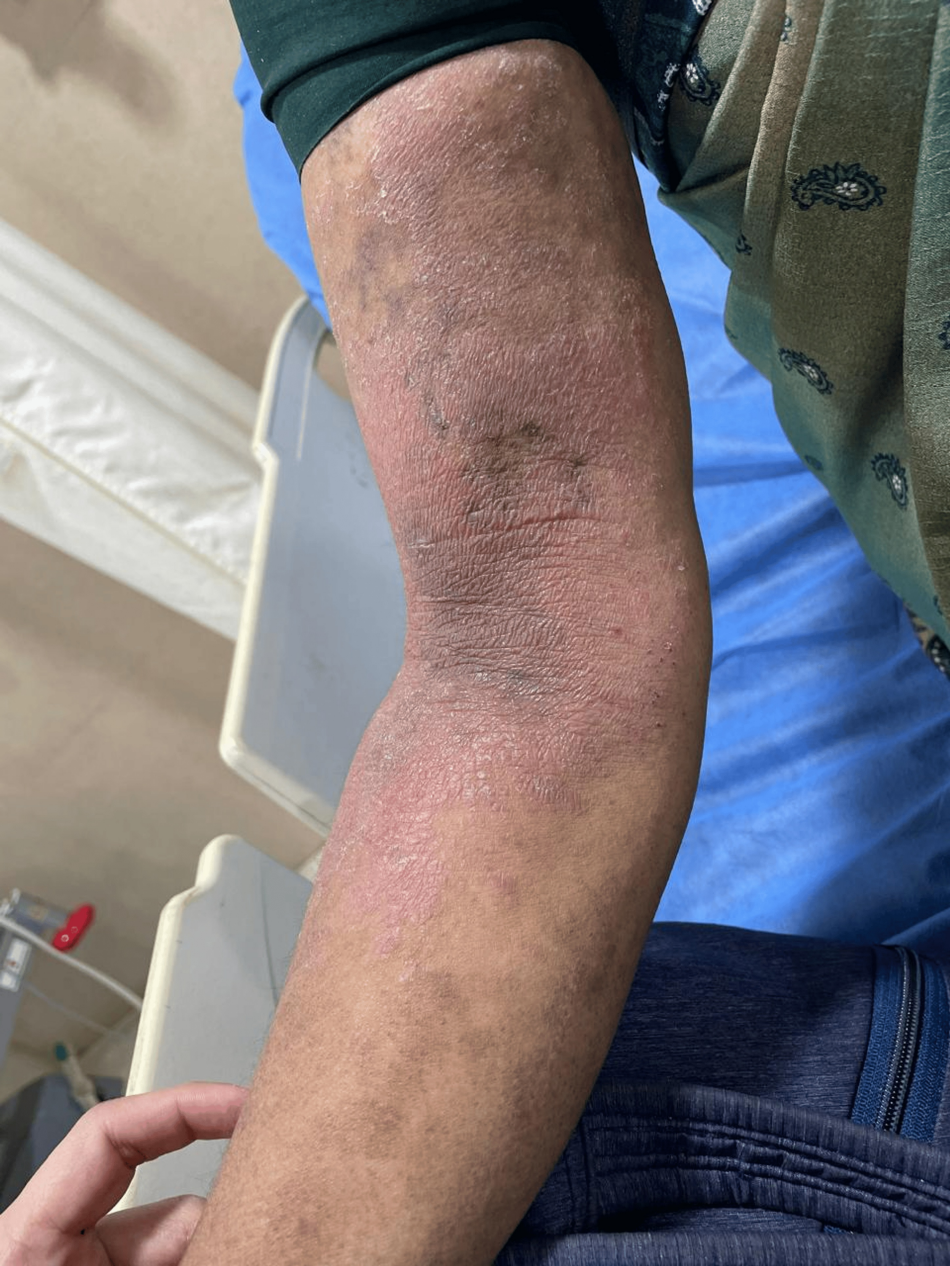
Erythematous and vesicular eczematous plaques involving the right antecubital fossa and extensor forearm overlying areas of chronic lichenification

**Figure 2:**
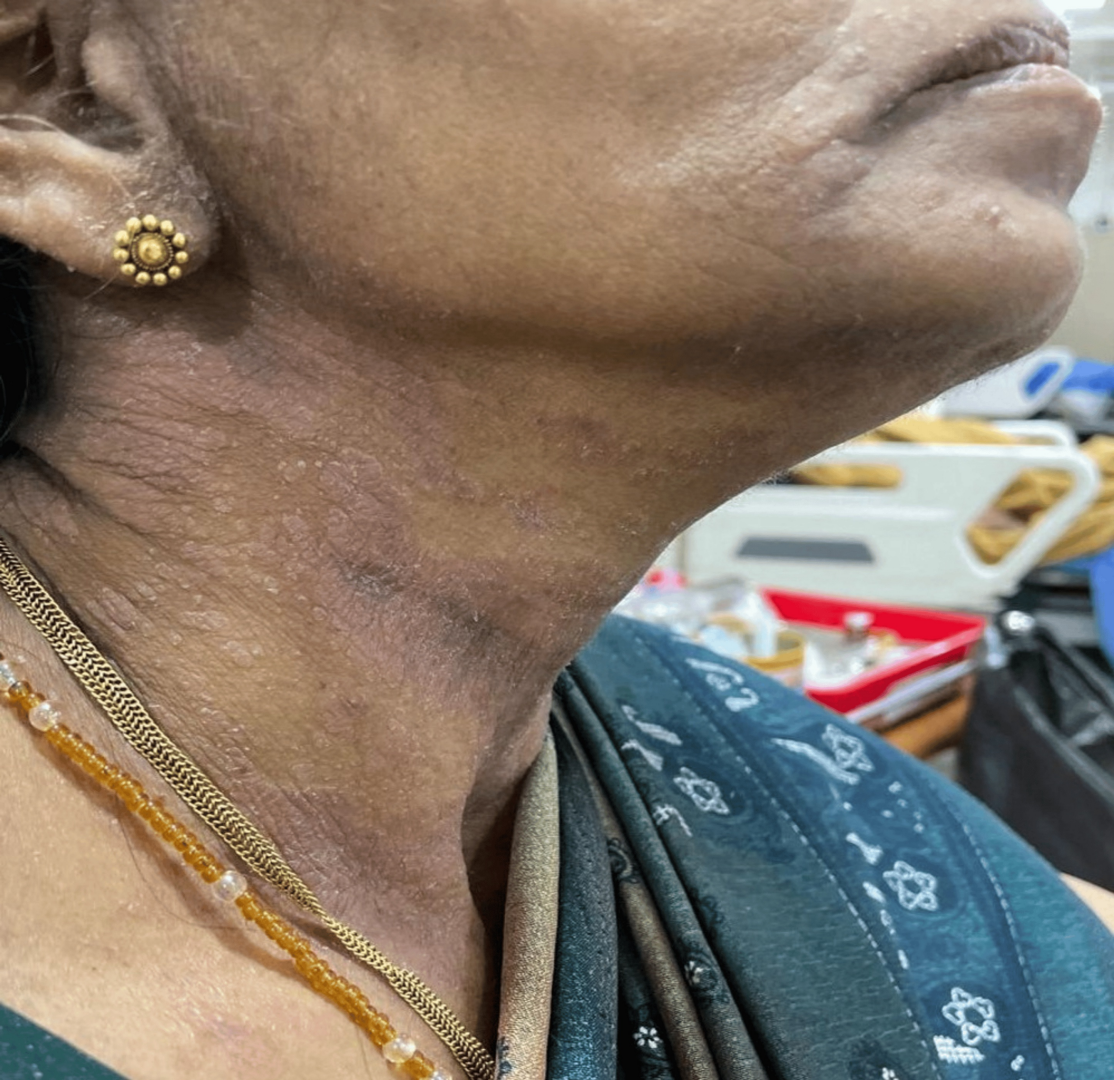
Erythematous, edematous plaques with scaling and scattered vesicles affecting the lower face and anterior neck in a V-distribution corresponding to uncovered skin

Dermatologic examination revealed chronic lichenified plaques on the dorsal hands with an acute superimposed eruption. Brightly erythematous, oedematous plaques with tightly clustered microvesicles were seen on the face (with pronounced periorbital swelling), the anterior neck, the V-shaped upper chest, and both extensor and flexural surfaces of the arms. Lesions were fixed, non-migratory, and sharply confined to exposed skin, with sparing beneath clothing (Figures 1, 2).

Given the severity of inflammation, she received an intravenous loading dose of methylprednisolone 125 mg, followed by 40 mg intravenously every 12 hours for 48 hours. Intravenous diphenhydramine 50 mg due to the severity of symptoms and H2 blockade (oral) was provided for symptomatic relief. She was admitted for dermatology-directed care.

Over 48 hours, her pruritus improved to 2/10, and facial swelling resolved. She was discharged on a 14-day oral prednisone taper (60 mg daily for three days, 40 mg daily for three days, 20 mg daily for four days, and 10 mg daily for four days). Clobetasol propionate 0.05% was prescribed for body lesions, and hydrocortisone butyrate 0.1% for facial involvement. She was counseled regarding complete avoidance of QAC disinfectants and advised to seek workplace modification. Patch testing was planned as an outpatient procedure, but could not be completed due to workplace constraints; however, the clinical pattern, chronicity, and temporal association strongly supported the ABCD diagnosis. The patient reported resolution of lesions via telephonic follow-up after 15 days.

## Discussion

This case illustrates the diagnostic difficulty posed by severe acute exacerbations of airborne ACD, particularly in sensitized individuals with chronic skin barrier dysfunction. Cleaning personnel are disproportionately affected due to repetitive exposure to aerosolized disinfectants, especially QAC-based products, which are both irritants and emerging allergens [3]. Re-exposure can precipitate intense T-cell activation and significant cutaneous inflammation. Repeated exposure to occupational irritants is a major risk factor, and airborne ACD is well documented in high-risk environments such as agriculture, construction, and healthcare settings [2,4]. Irritant exposures can also enhance sensitization by disrupting the epidermal barrier and amplifying the induction phase of ACD, thereby lowering the threshold for subsequent allergic reactions [5]. These mechanisms likely contributed to the abrupt and severe presentation observed in our patient.

The patient’s presentation required careful distinction from immunoglobulin E-mediated hypersensitivity. Although she exhibited pruritus and facial swelling, two key features separated the condition from anaphylaxis: stable hemodynamics and absence of respiratory or cardiovascular involvement [6]. In contrast to urticaria or angioedema (which cause fleeting wheals that shift in location), our patient had fixed erythematous plaques with vesicles restricted to exposed skin, supporting a contact hypersensitivity pattern [7,8].

Systemic corticosteroids are often required during severe flares to rapidly control the T-cell-mediated response. A gradual taper is important to minimize the risk of rebound inflammation [9]. Potent topical corticosteroids are useful for short-term control of severe inflammation on the body, whereas lower-potency agents are preferred for sensitive areas such as the face, as recommended in rational prescribing guidelines [10]. Antihistamines reduce pruritus, but do not address the underlying delayed hypersensitivity.

One limitation in this case is that patch testing could not be completed, which would have confirmed the exact allergen. While the clinical pattern and exposure history strongly suggested QAC-related airborne ACD, the lack of confirmatory testing leaves a small degree of uncertainty. This challenge is common in occupational cases, where access to testing and follow-up can be difficult. The case highlights the need for better availability of patch testing and workplace support for individuals regularly exposed to disinfectants. Long-term control depends on identifying and avoiding the triggering allergen and workplace mitigation, since continued exposure may lead to repeated severe episodes.

## Conclusions

This case demonstrates that acute exacerbation of airborne contact dermatitis should be considered when patients present to the emergency department with intense pruritus and facial swelling but maintain stable vital signs. Recognition of fixed, eczematous lesions confined to exposed skin helps differentiate delayed hypersensitivity from anaphylaxis or generalized urticaria. Rapid initiation of systemic steroids and appropriate topical therapy resulted in swift improvement. Long-term control depends on identifying and avoiding the triggering allergen, since continued exposure may lead to repeated severe episodes.
